# Spiritual well-being, surgical fear, and quality of recovery in patients undergoing abdominal surgery: a descriptive and correlational study

**DOI:** 10.1590/1516-3180.2026.3677.28072026

**Published:** 2026-09-21

**Authors:** Kezban Koraş Sözen, Özge Havva Birim

**Affiliations:** I Zubeyde Hanım Faculty of Health Sciences, Department of Surgical Nursing, Niğde Ömer Halisdemir University, Niğde, Türkiye.; II Zubeyde Hanım Faculty of Health Sciences, Department of Nursing, Niğde Ömer Halisdemir University, Niğde, Türkiye.

**Keywords:** Surgical Procedures, Operative, Postoperative Care, Spirituality, Fear, Holistic Nursing, Perioperative Psychological Responses, Spiritual Health Assessment, Patient-Centered Surgical Nursing, Mind-Body Dimensions of Recovery

## Abstract

**BACKGROUND:**

Despite the increasing emphasis placed on holistic care in healthcare, the impact of spiritual well-being on postoperative outcomes is unclear, particularly in patients who have undergone abdominal surgery. The postoperative period is characterized by significant physical and psychological stress.

**OBJECTIVE:**

To examine the relationship between spiritual well-being, surgical fear, and the quality of postoperative recovery in patients undergoing abdominal surgery.

**DESIGN AND SETTING:**

Descriptive and correlational study.

**METHODS:**

A total of 116 patients participated in the study. Data were collected using the Descriptive Characteristics Form, Three-Factor Spiritual Well-Being Scale, Surgical Fear Questionnaire, and Quality of Recovery-40 Questionnaire. We evaluated the relationships between patients’ levels of spiritual well-being, their fear of surgery, and the quality of postoperative recovery.

**RESULTS:**

Patients reported high spiritual well-being and low levels of surgical fear; the mean Quality of Recovery-40 Questionnaire score was 109.36 ± 8.52, below the theoretical midpoint of the scale. Correlation analysis revealed no statistically significant relationship between spiritual well-being and surgical fear (ρ = 0.02, p = 0.83) or postoperative quality of recovery (ρ = 0.12, p = 0.2). No significant relationship was found between surgical fear and quality of recovery (ρ = 0.11, p = 0.23).

**CONCLUSION:**

Spiritual well-being was not significantly associated with surgical fear or postoperative quality of recovery among patients undergoing abdominal surgery. These findings suggest that, although spirituality may contribute to patients’ internal coping processes, it may not directly influence immediate postoperative outcomes. Further studies involving diverse patient populations and longitudinal designs are needed to better understand the relationship between spirituality, psychological responses, and recovery outcomes in surgical care.

## INTRODUCTION

Globally, an estimated 313 million surgical procedures are performed each year, with the number of abdominal surgeries in developed countries increasing annually by approximately 2% to 5%.^1,2^ Abdominal surgeries involve operations on vital organs within the abdominal cavity and are among the most commonly performed surgical interventions. Despite remarkable advancements in surgical techniques, medical technology, and perioperative care, both major and minor complications are a reality for many patients undergoing these procedures.^1,2^

Innovations in surgical science have significantly reduced the risk of postoperative complications; however, the emotional burden of surgery—particularly preoperative anxiety—continues to affect a large proportion of patients. The anticipation of surgery, regardless of its complexity or level of risk, often triggers heightened anxiety. If not identified and appropriately managed, this anxiety can negatively influence postoperative recovery outcomes, including pain perception, healing, and overall satisfaction.^3,4^

A comprehensive preoperative assessment is essential, because many patients do not voluntarily disclose their fears unless directly asked. This evaluation plays a vital role in preparing patients psychologically for surgery and in identifying any concerns that may impact their recovery and overall well-being.^3,5,6^ Specifically in abdominal surgery, identifying the sources and intensity of preoperative fear can guide healthcare providers in delivering targeted and effective pre- and postoperative care. Surgical nurses, in particular, play a critical role in this process. By implementing education-based interventions designed to reduce anxiety, nurses embody a holistic approach that contributes meaningfully to improved surgical outcomes.^3,7,8,9^

One area that is gaining increased attention in this context is spirituality. Defined as a dynamic and personal journey through which individuals seek meaning, connection, and inner peace, spirituality has become a key pillar in holistic healthcare. It helps individuals better understand themselves, others, and their place in the world.^10^ Spiritual well-being is closely linked to mental health, resilience, and overall quality of life, and has been shown to enhance coping during stressful or traumatic events such as surgery. Studies suggest that spiritual care can improve both physical and psychological health outcomes, potentially accelerating recovery and enhancing patient satisfaction.^10,11,12,13^

Addressing the psychological and emotional needs of surgical patients is as critical as executing the technical aspects of care. Surgery does not occur in a vacuum; it intersects deeply with a patient’s inner world, their emotions, beliefs, values, and fears. A patient-centered approach to surgical care must therefore include attention to the spiritual and psychological dimensions of health. Neglecting these aspects can undermine the trust, communication, and shared decision-making that are vital to quality care.^14^

Despite the recognized importance of spiritual well-being in the broader healthcare context, there is a striking lack of research that specifically examines the relationship between spiritual well-being, surgical fear, and quality of recovery, particularly in patients undergoing abdominal surgery. In this study, we sought to address this gap by investigating the associations between spiritual well-being, surgical fear, and postoperative quality of recovery in patients who undergo abdominal surgery. By doing so, we aim to contribute new insights into how psychological and spiritual dimensions may or may not interact in the context of acute surgical care.

## METHODS

### Design

A descriptive correlation design was used in this prospective study. The Strengthening the Reporting of Observational Studies in Epidemiology checklist was followed to report the study process.^15^

### Participants

The study population consisted of patients who underwent abdominal surgery between May 2023 and May 2024, including all patients over the age of 18 who could communicate in Turkish and agreed to participate in the study. The sample comprised a total of 200 patients who met the inclusion criteria. Patients who withdrew consent, required intensive care after surgery, or declined to complete the survey questionnaires were excluded from the study. The sample size of the study was calculated using the G*Power 3.1.9.7 software. Accordingly, it was determined that 96 patients were needed, with a 95% confidence interval and 5% margin of error. However, considering the possibility that some patients might be excluded during the study, 116 patients were included.

### Data collection instruments

Data were collected using the Descriptive Characteristics Form, Three-Factor Spiritual Well-Being Scale (TFSWBS), Surgical Fear Questionnaire (SFQ), and Quality of Recovery-40 Questionnaire (QoR-40).

The Descriptive Characteristics Form, designed by the researcher in accordance with the literature,^1,2,10^ was used to collect information such as the patient’s age, sex, and educational background.

The TFSWBS was initially developed in 2017 by Ekşi and Kardaş,^16^ and named the Spiritual Well-Being Scale. However, to avoid confusion with another scale of the same name that was developed by Paloutzian and Ellison^17^ in 1982, it was renamed in 2019 to the Three-Factor Spiritual Well-Being Scale. The scale, consisting of 29 items, is used to evaluate three distinct subdimensions: transcendence, harmony with nature, and anomie. Transcendence refers to the recognition that one’s existence extends beyond the self, integrating with the larger whole. Harmony with nature denotes an individual’s maintenance of their intrinsic connection to the natural world. Anomie describes a state in which societal norms no longer effectively govern individual behavior, leading to uncertainty in conduct. Scores for each subdimension are derived by aggregating designated items: for transcendence, items 1, 4, 5, 8, 9, 12, 13, 16, 17, 20, 21, 24, 25, 27, and 29; for harmony with nature, items 2, 6, 10, 14, 18, 22, and 28; and for anomie, items 3, 7, 11, 15, 19, 23, and 26. Elevated scores within a subdimension indicate a greater presence of the trait in question. The total score is calculated by summing the scores of the 29 items after reverse-scoring the anomie items. Total scores range from 29 to 145, with higher scores indicating greater spiritual well-being.^16^

The SFQ was developed by Theunissen et al.^18^ in 2014 and validated for the Turkish population by Bağdigen and Özlü^19^ in 2018. This scale comprises eight items scored on a numerical scale ranging from 0 (*no fear*) to 10 (*extreme fear*). It is divided into two subscales, each containing four items that measure the fear associated with the short-term and long-term outcomes of surgery. Items 1 to 4 assess fears related to immediate surgical outcomes, while items 5 to 8 assess fears concerning the long-term consequences. The lowest possible score on the scale is 0, and the highest is 80. Higher scores on the SFQ indicate higher levels of fear.

The QoR-40 was developed by Myles et al.^20^ in 2000 and validated in Turkish by Karaman et al.^21^ in 2014. The questionnaire is a comprehensive 40-item measure of postoperative recovery. It includes the five subdimensions of emotional state, physical comfort, patient support, physical independence, and pain. Each item is scored from 1 to 5. Subdimension scores are calculated by summing the relevant items, and the total score is obtained by summing all 40 items. The total score ranges from 40 to 200, with higher scores indicating better postoperative recovery. Because the QoR-40 does not have a universally accepted clinical cutoff for low, moderate, or high recovery, scores should be interpreted according to the theoretical range, scale midpoint, subdimension profile, timing of assessment, and comparisons with previous studies.

### Data collection

Data were collected by the researcher through face-to-face interviews. The TFSWBS and SFQ were administered during the preoperative period, whereas the QoR-40 was administered in the early postoperative period. The early postoperative period can be a few hours after short and simple surgeries, or it can extend to up to 24 hours after long and complicated surgeries.^22^ In this study, the early postoperative period was defined as the second hour after surgery, and the instruments were administered exactly 2 hours after surgery.

### Ethical considerations

The study was carried out in accordance with the principles of the Declaration of Helsinki. The study was started after approval was obtained from the Niğde Ömer Halisdemir University Ethics Committee (meeting date, April 3, 2023; decision number, 2023/06-03) and permission was granted by the institution where the study was conducted. Both written and verbal consent were obtained from all participants. Furthermore, it was communicated to the patients that their involvement in and the outcomes of the study would have no impact on their medical care. The data collection instruments did not solicit identifying information such as names, surnames, or protocol numbers, ensuring that all data were gathered anonymously.

### Data analysis

The analysis of the data was conducted using IBM SPSS Statistics for Windows version 24.0 (IBM Corp., Armonk, New York), using frequency distributions for categorical variables and descriptive statistics for numerical variables. The normality of the data distribution was assessed using the Kolmogorov–Smirnov test. Because the data did not follow a normal distribution, nonparametric tests were used: the Mann–Whitney U test for pairwise comparisons and Kruskal–Wallis analysis of variance test for multiple comparisons. To examine the relationships between variables, Spearman’s correlation analysis was performed. A p value of less than 0.05 was considered statistically significant.

## RESULTS

The average age of the patients was 52.61 ± 16.8 years, with 51.7% being male and 82.8% undergoing surgery under general anesthesia (**Table 1**). Of the patients, 32.8% had hernia surgery, 69% had previously undergone surgery, 78.4% were married, 85.3% had children, 50.9% had an educational background of primary school, 70.7% had an income that covered their expenses, and 56% did not have any chronic diseases.

**Table 1:** Comparison of patients’ descriptive characteristics and instrument scores

Variable	Number (%)	Instrument score Mean ± SD		
		TFSWBS	SFQ	QoR-40
Sex				
*Female*	56 (48.3)	120.69 ± 7.10	17.85 ± 11.93	109.32 ± 8.64
*Male*	60 (51.7)	120.7 ± 9.7	10.25 ± 10.65	109.40 ± 8.49
*Test = MWU*		−0.188	−4.052	0.634
*p*		0.85	**0**	0.52
Type of anesthesia				
*General*	96 (82.8)	120.23 ± 7.87	14.53 ± 11.89	109.02 ± 8.64
*Spinal*	20 (17.2)	122.90 ± 11.06	11.00 ± 11.58	109.40 ± 8.49
*Test = MWU*		−0.15	−1.512	0.439
*p*		0.88	0.13	0.66
Type of surgery				
*Hernia*	38 (32.8)	122.15 ± 9.05	12.21 ± 11.09	108.93 ± 8.84
*Laparoscopic Cholecystectomy*	29 (25)	121.20 ± 6.56	14.24 ± 9.08	108.10 ± 7.97
*Bariatric surgery*	7 (6)	113 ± 14.23	10 ± 10.37	110.28 ± 9.12
*Colon cancer*	4 (3.5)	118.5 ± 3.1	18.75 ± 24.73	112.25 ± 15.32
*Stomach cancer*	14 (12)	122.64 ± 7.53	17.64 ± 17.5	106.35 ± 6.12
*Appendectomy*	16 (13.8)	117.37 ± 9.09	10.2 ± 11.35	113.62 ± 8.62
*Ileus*	8 (6.9)	123 ± 3.46	10.75 ± 6.18	110.93 ± 7.1
*Test = KW*		13.809	4.221	7.312
*p*		**0.03**	0.64	0.29
Previous experience of surgery				
*Yes*	80 (69)	120.93 ± 8.86	14.41 ± 12.65	109.31 ± 9.29
*No*	36 (31)	120.16 ± 7.78	12.83 ± 9.98	109.47 ± 6.3
*Test = MWU*		−0.395	−0.32	0.932
*p*		0.69	0.74	0.35
Marital status				
*Married*	91 (78.4)	121.79 ± 7.98	14.24 ± 11.83	109.20 ± 7.95
*Single*	25 (21.6)	116.72 ± 9.34	12.76 ± 12.16	109.92 ± 10.53
*Test = MWU*		−3.027	−0.797	0.437
*p*		**0**	0.42	0.66
Do you have children				
*Yes*	99 (85.3)	121.39 ± 8.1	13.59 ± 11.66	108.35 ± 8.06
*No*	17 (14.7)	116.72 ± 9.34	15.82 ± 13.22	115.23 ± 9.03
*Test = MWU*		−2.376	0.571	0.34
*p*		**0.01**	0.56	0.51
Educational background				
*Illiterate*	15 (12.9)	120.8 ± 7.82	14.66 ± 6.85	101.66 ± 8.85
*Primary school*	59 (50.9)	123.54 ± 7.54	12.13 ± 12.13	110 ± 7.58
*High school*	21 (18.1)	119.47 ± 5.62	14.9 ± 13.72	118 ± 7.83
*University*	21 (18.1)	113.85 ± 10.11	17.42 ± 11.77	113.71 ± 8.33
*Test = KW*		22.148	5.501	1.659
*p*		**0**	0.13	0.18
Economic status				
*Income is less than expenses*	34 (29.3)	122.35 ± 6.19	15.41 ± 14.72	108.58 ± 10.12
*Income covers expenses*	82 (70.7)	120.01 ± 9.25	13.30 ± 10.5	109.68 ± 7.81
*Test = MWU*		−2.321	−0.343	0.307
*p*		**0.02**	0.73	0.75
Chronic disease status				
*Yes*	51 (44)	121.62 ± 7.41	13.19 ± 9.97	107.64 ± 8.34
*No*	65 (56)	119.96 ± 9.28	14.49 ± 13.22	110.70 ± 8.49
*Test = MWU*		−2.223	0.011	1.382
*p*		**0.02**	0.99	0.16
Age (mean ± SD)	52.61 ± 16.8			

KW, Kruskal–Wallis analysis of variance test; MWU, Mann–Whitney U test; QoR-40, Quality of Recovery-40 Questionnaire; SD, standard deviation; SFQ, Surgical Fear Questionnaire; TFSWBS, Three-Factor Spiritual Well-Being Scale. Bold values indicate statistically significant results (p < 0.05).

When examining the average instrument scores in terms of sex, the SFQ score for female(17.85 ± 11.93) was significantly higher than that for male (10.25 ± 10.65). The average scores for the TFSWBS and QoR-40 showed no significant sex-based differences.

When examining the average instrument scores of patients in terms of the type of surgery, a statistically significant difference was found in the TFSWBS scores. No significant differences in type of surgery were detected in the average SFQ and QoR-40 scores.

From the perspective of marital status, a review of the average instrument scores showed that married individuals had a higher average TFSWBS score (121.79 ± 7.98) than those who were single, and that this difference was statistically significant. No significant difference in marital status was found in the SFQ and QoR-40 scores.

The average instrument scores were reviewed in terms of having children. Patients with children had higher TFSWBS scores (121.39 ± 8.1) than those without, with this difference being statistically significant. No significant differences were observed in the average SFQ and QoR-40 scores when they were analyzed according to having children.

In terms of educational background, examining the average instrument scores of patients revealeda statistically significant difference in the TFSWBS scores. No significant differences were found in the average SFQ and QoR-40 scores when they were analyzed by educational status.

Reviewing the average instrument scores from an economic standpoint showed that patients with a lower income level had a higher average TFSWBS score (122.35 ± 6.19) than those with a higher level, and this difference was statistically significant. No significant differences were found in the average SFQ and QoR-40 scores when considering economic status.

For chronic illness, a higher average TFSWBS score (121.62 ± 7.41) was found in patients with a chronic illness than in those without; this difference was statistically significant. No significant differences were observed in the average SFQ and QoR-40 scores in terms of chronic illness. When the average instrument scores of patients were examined in terms of the type of anesthesia and previous surgical experience, no significant difference was found in the TFSWBS, SFQ, and QoR-40 scores.

The mean total TFSWBS score was high (120.69 ± 8.51; **Table 2**). The transcendence (73.18 ± 7.01) and harmony with nature (33.11 ± 2.8) subdimension scores were high, whereas the anomie score was low (14.39 ± 4.31). The mean total QoR-40 score was 109.36 ± 8.52. This score was below the theoretical midpoint of 120 on the scale’s score range of 40 to 200, and was therefore interpreted as reflecting comparatively limited recovery at the second postoperative hour; this interpretation does not represent a validated diagnostic cutoff. The scores for physical independence (20.68 ± 3.66) and patient support (30.24 ± 2.22) were higher, whereas the physical comfort, emotional state, and pain scores were lower relative to their possible ranges. The mean total SFQ score was low (13.92 ± 11.87), and both the short-term and long-term fear subdimension scores were also low.

**Table 2:** Patients’ mean total and subdimension total scores for each instrument

Instrument and subdimension	Mean ± SD	Min–Max
TFSWBS total score	120.69 ± 8.51	(29–145)
*Transcendence*	73.18 ± 7.01	(15–75)
*Harmony with nature*	33.11 ± 2.8	(7–35)
*Anomie*	14.39 ± 4.31	(7–35)
QoR-40 total score	109.36 ± 8.52	(40–200)
*Physical comfort*	26.71 ± 4.4	(12–60)
*Emotional state*	20.71 ± 2.84	(9–45)
*Physical independence*	20.68 ± 3.66	(5–25)
*Patient support*	30.24 ± 2.22	(7–35)
*Pain*	11.00 ± 3.11	(7–35)
SFQ total score	13.92 ± 11.87	(0–80)
*Short term*	10.05 ± 7.4	(0–40)
*Long term*	3.87 ± 6.32	(0–40)

Max, maximum; Min, minimum; QoR-40, Quality of Recovery-40 Questionnaire; SD, standard deviation; SFQ, Surgical Fear Questionnaire; TFSWBS, Three-Factor Spiritual Well-Being Scale.

No statistically significant relationship was found between the patients’ average TFSWBS, SFQ, and QoR-40 scores (**Table 3**).

**Table 3:** Relationships between patients’ mean instrument scores

		TFSWBS	SFQ	QoR-40
TFSWBS	ρ	1	0.02	0.12
	p		0.83	0.2
SFQ	ρ	0.02	1	0.11
	p	0.83		0.23
QoR-40	ρ	0.12	0.11	1
	p	0.2	0.23	

QoR-40, Quality of Recovery-40 Questionnaire; SFQ, Surgical Fear Questionnaire; TFSWBS, Three-Factor Spiritual Well-Being Scale,ρ, Spearman’s rank correlation coefficient.

## DISCUSSION

In this study, we investigated the relationship between the level of spiritual well-being, surgical fear, and quality of recovery in patients who underwent abdominal surgery; we established that patients’ levels of spiritual well-being were high. A study by Çiçekli and Çalışkan,^23^ as well as one by Sandau et al.,^24^ also identified high levels of spiritual well-being. Conversely, the study by Çınar and Bülbüloğlu^25^ found that patients’ levels of spiritual well-being were low. These differences may be attributed to the subjective nature of spiritual well-being, which can vary from individual to individual and overtime. Furthermore, spiritual well-being is influenced by cultural, social, and individual belief systems, which may lead to variability in findings across different populations and healthcare settings.

The findings of this study revealed that patients who have undergone gastric cancer surgery, are married and have children, are illiterate, have a low income level, and have chronic diseases have high levels of spiritual well-being. By contrast, studies conducted by Bezerra et al.^26^ and by Çiçekli and Çalışkan^23^ showed no significant correlation between the level of spiritual well-being and marital status, educational level, having children, or income level. The higher correlations found in the present study may be attributed to the complication and mortality rates associated with major surgeries such as those for gastric cancer. Facing life-threatening treatment for a condition, such as surgery for cancer, may increase individuals’ need to seek meaning, hope, and spiritual resources; this may explain the elevated spiritual well-being scores observed in these patients.

Married patients with children who have hopes and plans may also be considered to have elevated levels of spiritual well-being. Similarly, individuals with low levels of education and income may have higher levels of spiritual well-being due to a tendency to cling more to spiritual beliefs in the pursuit of well-being; individuals with chronic illnesses may turn to religion as a life purpose, thereby increasing their levels of spiritual well-being; and chronic illness may prompt individuals to engage more deeply with existential and spiritual questions, potentially strengthening their reliance on spiritual coping strategies.^23,24,25,26,27^

We found no significant difference in the levels of spiritual well-being between male and female patients. Similarly, studies by Çiçekli and Çalışkan^23^ as well as by Çınar and Bülbüloğlu^25^ identified no differences in spiritual well-being between sex. Conversely, the study by Yaghoobzadeh et al.^27^ found that female had higher levels of spiritual well-being, whereas the study by Dehbashi et al.^28^ found that male had higher levels. These differences may stem from cultural variations and the ways in which spirituality is learned. Sex differences in spirituality reported in the literature may also reflect differences in social roles, coping styles, and culturally shaped expressions of religiosity and spirituality.

In this study, we observed that patients’ levels of surgical fear are low. The studies by Işıklı et al.,^29^ Demirci and Şahin,^30^ and Theunissen et al.^18^ also reported low levels of surgical fear. By contrast, the study by Satır et al.^31^ indicated a moderate level of fear. The variation in surgical fear scores may be due to personal characteristics, the magnitude of the surgical operation, the type of anesthesia administered, differences in complications, and the patient’s family and social support mechanisms. Preoperative education, previous surgical experiences, and trust in healthcare professionals may also contribute to differences in perceived surgical fear among patients.

We determined that female experience greater surgical fear than men. Similarly, Satır et al.^31^ and Çağlar and Özlü^32^ found that women’s scores for surgical fear were higher. This can be explained by the heightened emotional disposition of female, the fear of not being able to reunite with their children due to the maternal role, and men’s tendency to express their fear less than female do. Another possible explanation is that female may report and express health-related anxieties more openly, whereas male may underreport fear due to sociocultural expectations regarding emotional expression.

This study’s findings show that the type of surgery patients undergo, their marital status, whether they have children, as well as their levels of education and income, do not influence the level of surgical fear. The findings of the study are consistent with the data in the literature.^29,31^ These results suggest that surgical fear may be more strongly associated with individual psychological factors than with sociodemographic characteristics.

The mean score for quality of recovery observed in this study was lower than the scores reported by Demirci and Şahin^30^ and Gümüş.^3^ The QoR-40 has a theoretical score range of between 40 and 200, with higher scores indicating better recovery. However, the instrument does not have a universally accepted cutoff for categorizing recovery as low, moderate, or high. Therefore, the mean score of 109.36 was interpreted cautiously as comparatively limited because it was below the theoretical midpoint of 120 and because lower scores were observed particularly in the physical comfort, emotional state, and pain domains. Differences from previous studies may be related to the patient population, type and magnitude of surgery, surgical technique, institutional care practices, and, importantly, the timing of assessment. In the present study, the QoR-40 was administered at the second postoperative hour, when pain, residual anesthetic effects, fatigue, restricted mobility, and psychological stress may still have been prominent. Accordingly, the score should be understood as an indicator of very early postoperative recovery rather than of the patient’s overall recovery trajectory.

We determined in this study that the sex of patients, the type of surgery they undergo, their marital status, whether they have children, their levels of education and income, and whether they have a chronic illness do not affect the quality of recovery. No significant relationship was found between the levels of spiritual well-being of patients undergoing abdominal surgery and the levels of surgical fear and quality of recovery. Similarly, the study by Çınar and Bülbüloğlu^25^ found no meaningful relationship between spiritual well-being and surgical fear. In contrast to the findings of this study, some findings in the literature indicate that spiritual well-being positively affects the recovery process.^33,34^ One possible explanation for this finding is that spiritual well-being may influence long-term psychological adaptation and coping rather than immediate postoperative clinical outcomes such as the quality of early recovery or acute surgical fear. Furthermore, the relatively high mean score observed for spiritual well-being in this study may have limited variability in the data, potentially reducing the likelihood of detecting significant correlations.

### Limitations

First, the relatively high level of spiritual well-being and low level of surgical fear observed among participants may have reduced score variability and limited the ability to detect statistically significant relationships. Second, the study was conducted with patients undergoing abdominal surgery at a single institution, which limits the generalizability of the findings. Third, postoperative quality of recovery was assessed only once, at the second postoperative hour. This very early measurement may have been influenced by residual anesthesia, acute pain, fatigue, and temporary mobility restrictions and may not fully reflect changes in the quality of recovery during later postoperative stages. Fourth, the cross-sectional correlational design prevents causal inferences from being made regarding the relationships among spiritual well-being, surgical fear, and quality of recovery. Future multicenter studies should include larger and more diverse samples and repeated QoR-40 assessments at later postoperative time points to examine changes in recovery over time.

## CONCLUSION

The findings of this study indicated that patients undergoing abdominal surgery reported high levels of spiritual well-being and low levels of surgical fear. Their mean QoR-40 score was below the theoretical midpoint, suggesting comparatively limited recovery at the second postoperative hour rather than a clinically validated category of poor recovery. However, no statistically significant relationship was found between spiritual well-being and either surgical fear or postoperative quality of recovery. A possible explanation for this outcome may lie in the acute nature of abdominal surgical procedures, which—despite being invasive—typically involve short-term recovery processes and intensive perioperative care, differing from chronic health conditions in which long-term spiritual coping mechanisms may have a stronger influence on patient outcomes. Although the associations were not statistically significant in this population, these results may provide valuable insights when compared with findings from studies involving other types of surgical procedures, different cultural contexts, or chronic disease populations. For healthcare professionals, particularly nurses, these findings highlight that, although spiritual well-being may not directly influence measurable postoperative outcomes, spiritual care remains an important component of holistic and patient-centered care. Future research involving diverse patient populations and longitudinal designs are essential to further explore the complex interplay between spirituality, psychological responses, and recovery outcomes in surgical care.

## Data Availability

Data supporting the findings of this study are available upon reasonable request from the corresponding author, Kezban Koraş Sözen.
